# What they fill in today, may not be useful tomorrow: Lessons learned from studying Medical Records at the Women hospital in Tabriz, Iran

**DOI:** 10.1186/1471-2458-8-139

**Published:** 2008-04-27

**Authors:** Faramarz Pourasghar, Hossein Malekafzali, Alireza Kazemi, Johan Ellenius, Uno Fors

**Affiliations:** 1 Department of Learning, Informatics, Management and Ethics (LIME), Karolinska Institute, Stockholm, Sweden; 2 Tabriz University of Medical Sciences, Tabriz, Iran; 3 Faculty of Public Health, Tehran University of Medical Sciences, Tehran, Iran; 4 National Public Health Management Center (NPMC), Iran

## Abstract

**Background:**

The medical record is used to document patient's medical history, illnesses and treatment procedures. The information inside is useful when all needed information is documented properly. Medical care providers in Iran have complained of low quality of Medical Records. This study was designed to evaluate the quality of the Medical Records at the university hospital in Tabriz, Iran.

**Methods:**

In order to get a background of the quality of documentation, 300 Medical Records were randomly selected among all hospitalized patient during September 23, 2003 and September 22, 2004. Documentation of all records was evaluated using checklists. Then, in order to combine objective data with subjective, 10 physicians and 10 nurses who were involved in documentation of Medical Records were randomly selected and interviewed using two semi structured guidelines.

**Results:**

Almost all 300 Medical Records had problems in terms of quality of documentation. There was no record in which all information was documented correctly and compatible with the official format in Medical Records provided by Ministry of Health and Medical Education. Interviewees believed that poor handwriting, missing of sheets and imperfect documentation are major problems of the Paper-based Medical Records, and the main reason was believed to be high workload of both physicians and nurses.

**Conclusion:**

The Medical Records are expected to be complete and accurate. Our study has unveiled that the Medical Records are not documented properly in the university hospital where the Medical Records are also used for educational purposes. Such incomplete Medical Records are not reliable resources for medical care too. Some influencing factors external to the structure of the Medical Records (i.e. human factors and work conditions) are involved.

## Background

A Medical Record is a set of information about health status of a patient which acts as a media to establish effective communication within a treatment provider team [1-3]. It is a source of important medical data and information which presents medical history and status of the patient and also have educational and research value [4]. Furthermore, the Medical Record can also be used as a tool for follow-up and supervising treatment procedures in the community.

The information which is documented on the Medical Records must be accurate, valid and updated. The documentation task is usually performed by physicians, nurses and/or clerical staff and the whole treatment provider team has responsibility to secure the accuracy of the record.

Since the input of information is performed by different persons, at different points of time, and because it is often done after the medical service has been administered, the information sometimes is not as precise as it should be expected. The documenter might for instance forget to document some informational elements intentionally or unintentionally; forget to register time and date, and/or sign the document at the end of documentation process. These kinds of problems might affect the quality of medical care and are in contrast with the main goal of a medical record, as being a reliable source of information [5]. Several studies have evaluated Medical Records from different points of view, and have found quality problems of various kinds [6].

In Iran, almost all medical care institutions are using Paper-based Medical Records (PBMR) as the primary source of medical information [7]. Authorities at the Medical Universities and also at other institutions which use information from the Medical Records (i.e. Insurance companies, Forensic/Legal medicine, Courts) have always recommended that all Medical Records must be complete and accurate. However they often report a lack of information in the Medical Records they have evaluated from hospital records [8].

The aim of this study was to evaluate the quality of documentation of PBMR in terms of completeness, availability and usability at the Women Hospital of Tabriz University of Medical Sciences, Iran. Since it had earlier been indicated that some of the Medical Records were incomplete, but there was no exact figures of completeness/incompleteness of Medical Records at the university hospitals, this study focused on drawing a general picture of the quality of the Medical Records system at the university hospital, and then to measure the completeness/incompleteness of Medical Records and finally to find out the probable reasons of shortcomings in Medical Records. On the other hand, the quality of Medical Records had never been seen from the physicians and nurses view points. This study also aimed to evaluate opinions of medical staff on the quality of Medical Records system as the main providers and consumers of the medical information. A further aim was to collect background information to serve as a base for assessment of possible advantages of introducing Electronic Medical Records (EMR) systems in Iran.

## Methods

This study is a retrospective-descriptive study on the data gathered from Medical Records of hospitalized patients at the Women hospital during a 12 month period from September 23, 2003 through September 22, 2004.

In the Iran medical care system, there are several hospitals, including public hospitals which are run by the Ministry of Health and Medical Education of Iran (MOHME) through universities, private hospitals which are run by private sector physicians and social insurance hospitals which are run by social insurance organization. The university hospitals are dominant in terms of number of the hospitals, number of beds, variety of specialties and services they provide. The university hospitals are public hospitals and available for all people. Patients are usually admitted through the hospitals' clinics or are referred from other health centers and/or private sector physicians.

The patients are required to pay the hospitalization expenses, and if the patients have contract with insurance companies, this companies cover the expenses. Several insurance companies are active in the Iran medical care system.

The women hospital, Alzahra, is the second largest university hospital in Tabriz and is located downtown. This hospital provides medical care for about 20,000 inpatients each year. The PBMR system is the only available documentation system at the hospital. No extra records are kept at the wards. There is an index book at the Medical Records department for recording the identification information of patient, record unit number and the diagnosis after discharge. This book is used to retrieve patient's Medical Records. All information regarding the treatment of patient is written directly by hand on the sheets. Each record is kept at the Medical Records archive at the hospital and is retrieved for use when the patient is admitted for inpatient care or returned to the outpatient clinic for follow up.

A typical Medical Record in the Iran medical care system contains a set of sheets including: admission and discharge summary, medical history and physical examination, physician's order, progress note, laboratory report attachment, radiology report, electrocardiogram attachment, consultation request, vital signs, composite graphic chart, fluid balance chart (24 hours), pre-operation care, anesthesia record, operation report, pathology report and unit summary sheet.

On every sheet there are predefined places for documentation of information regarding identification of patient and physician, clinical exams, medical or surgical interventions and a summary report of Medical Records when the patient is discharged from the hospital. The MOHME has published a set of standard formats for the Medical Records at the University hospitals, which clarify shape, format and content of each sheet of the Medical Records. Based on these standards, the sheets on the Medical Records are categorized in two groups. Each Medical Record comprises a set of fixed and essential sheets. These sheets are mandatory on every record. These are: admission and discharge, medical history and physical examination, physician's order, progress note, laboratory report attachment, vital signs, composite graphic chart and unit summary sheet.

The next group is those sheets that are added based on needs and medical status of the patient. These sheets are: radiology report, electrocardiogram attachment, consultant request, fluid balance (24 hours), pre operation care sheet, anesthesia record, operation report, and pathology report sheet.

Among the 19803 Medical Records of hospitalized patients at the Women hospital between September 23, 2003 and September 22, 2004, 300 Medical Records were randomly selected using an individual unit number which were registered in the index book. All selected records were available immediately.

Since each sheet of the Medical Record contains different informational elements and in order to facilitate the interpretation of the results; we categorized these informational elements into four groups:

A – Demographic information (including unit number, patient's name and family name, father name, date of birth, location of birth, address and phone number)

B – Administrative information – admission information (including date of admission, admitting physician, ward, room and bed number)

C – Diagnostic and treatment procedures (including physical examination, laboratory and radiological tests, medical orders and surgical interventions)

D – Identification information of diagnosis and treatment provider (name and family name of physician and nurse, signature, seal, date and time)

Three aspects of potential problems were studied: availability, completeness and ease of use of Medical Records.

### Availability

The presence or absence of any required sheet was determined per patient as simple test on availability. Every selected Medical Records was checked if the essential sheets existed; and also when the clinical condition of the patient had mandated to use additional sheets, did those sheets exist in the medical record.

### Completeness

A set of 16 checklists were designed (one for every sheet of medical record) for evaluating the content of records in terms of compatibility with recommended standard format, completeness of medical information, date, time, name and signature of documenter. The checklists were based on the standard Medical Record at the university hospitals in Iran. In these checklists, there was a place for every requested item on every medical record sheet. If the requested information was registered in the sheets correctly, a check mark was consequently placed in the checklist for that specific item. Generally the requested information on each sheet included identification information of patient, physician and ward, the result of medical or surgical interventions and/or laboratory and radiological tests and finally date, time and signature of the care provider.

Since it is expected that the Medical Records hold all clinical information of the patient, the golden standard was to document all requested information elements in the records. Before collecting data and in order to perform a quality control on the checklists, a limited pilot test was carried out on the Medical Records to verify that the checklists covered all essential information.

### Ease of use

The ease of use and supplemental information on the possible problems with the existing Medical Records was investigated through interview with staff at the hospital. Ten physicians and ten nurses who were involved in documentation of Medical Records were randomly selected for interviewing. For this purpose, an alphabetically sorted list of all physicians and nurses who were working in the hospital was obtained from the hospital administration. Then ten physicians and ten nurses were selected using simple random sampling method. They were asked if they voluntarily would accept to be interviewed.

The physicians and the nurses were interviewed by using separate semi structured guidelines. Most of the interviews (based on consent of the interviewee) were recorded and then transcribed for analysis. This study was approved by the medical research ethics board in Iran.

## Results

### Availability: Presence or absence of mandatory sheets (for all groups)

Almost all records contained the essential sheets. Since the radiological exams, surgery, anesthesia, pathological exams and lab tests are requested based on the condition of the patient, the evaluation was only done on those records that were expected to have these actual sheets. Some sheets were missed; the highest number of missing belonged to the progress note sheet. More than 10% of the Medical Records were without progress note sheet (Table 1).

**Table 1:** The results of evaluation of 300 medical records in terms of availability and completeness at the Alzahra hospital, Tabriz, Iran (23/9/2003 – 22/9/2004)

Sheets	Number of existing sheets	Expected number of sheets	Group A * (%)	Group B † (%)	Group C ‡ (%)	Group D §(%)
Admission and discharge summary	300	300	71	78	88	81
Medical history & physical examination	300	300	67	73	91	100
Physician's order	299	300	54	72	98	100
Progress note	269	300	54	74	99	100
Laboratory report attachment	289	300	56	72	100	100
Radiology report	19	19	57	24	53	95
Electrocardiogram attachment	23	23	65	72	39	15
Consultation request	47	47	64	63	98	56
Vital signs	290	300	59	57	89	100
Composite graphic chart	292	300	57	57	51	N/A¶
Fluid balance chart	85	85	52	57	90	N/A¶
Pre-operation care	123	128	67	71	56	94
Anesthesia record	127	128	97	61	50	99
Operation report	128	128	94	60	69	98
Pathology report	50	50	95	56	51	22
Unit summary	300	300	99	61	87	98

* Percentage of the documentation of demographic information: Unit number, Patient's Name and Family name, Father Name, Date of Birth, Location of Birth, Address and phone number.

† Percentage of the documentation of administrative information: Date of admission, admitting Physician, Ward, Room and Bed number.

‡ Percentage of the documentation of diagnostic and treatment Procedures: Physical examination, Laboratory and Radiological exams, Orders, Medical and Surgical interventions.

§ Percentage of the documentation of identification information of diagnosis and treatment provider: Name and Family name of Physician and Nurse, Signature, Seal, Date and Time.

¶ It is not required to document identification information of care providers on these sheets.

### Completeness

#### a. Documentation of patient's identification (demographic) information (group A)

Each sheet should contain identification information (name, family name, and date of birth) on the header, and additional elements on some sheets. Documentation of this information varied on each sheet (Table 1), the unit summary sheet with the highest value of documentation (99%) and the Fluid balance chart with lowest value of documentation (52%).

#### b. Documentation of administrative information (group B)

Admission data are important in terms of accessing to patients' record and also for administrating purposes. On many of the records, this information was incomplete (Table 1). The highest value of documentation belonged to the admission and discharge sheet (78%).

#### c. Documentation of diagnostic and treatment Procedures (group C)

The contents of these sheets are important from medical point of view.

The percentage of documentation of these elements varied from as low as 39% on Electrocardiogram attachment sheet to as high as 100% on sheets which were filled in by physicians (Table 1).

#### d. Documentation of Identification information of diagnosis and treatment provider (group D)

Identification information of treatment providers are important in terms of legal issues and follow up of the patient. On essential sheets' group, most of this information had been documented well, in particular those sheets which had been filled in by physicians, such as the medical history and physical exam sheet, physician's order sheet and progress note all with 100% completeness of documentation of information (Table 1).

### Ease of use and general problems (Interview)

Ten physicians comprised of Obstetrics and Gynecologists, Anesthesiologists and Pediatricians were interviewed using a semi structured interview guideline. Also ten nurses were interviewed using a separate semi structured interview guideline.

The average work experience of the interviewed physician group at the hospital was 13.4 years and the nurses group was 16.9 years. The average of time that physicians had spent on documentation was 1 hour and 45 minutes of a working shift (8 hours). The nurses expressed that most of their working hours were spent for documentation, in average 15 minutes for each record.

Sixty percent of the interviewed physicians in response to the question "Have you documented all informational elements on related sheet in Medical Records" responded that they usually completed some parts of the records and 40% of physicians told that they have documented all requested informational elements. 60% of interviewed nurses reported that they have documented all informational elements because the insurance companies had put pressure on completeness of records. The rest of interviewed nurses told that they have never filled in all parts of the Medical Records because of too high workload. Almost all nurses believed that the Medical Records of patients, who had been admitted during afternoon and night shifts, were incomplete. The reason was that at the afternoon and night shifts, there was no clerical staff at the ward and nurses were forced to document header of all sheets and sometimes because of too high workload, this was not done.

When asked "Was it easy to get information out of the paper based Medical Records at the hospital"; 60% of physicians told that it was difficult and 40% told that it was easy for them to get the needed information. 80% of the interviewed nurses believed that retrieving of information from paper medical record was difficult; in contrast 20% believed that it was easy for them to get the needed information.

Ninety percent of the interviewed physicians considered poor handwriting as the main problem of the PBMR and 10% of the interviewees considered missing of sheets or incompleteness of informational element on sheet as the main problem of records. In the nurses' group, almost all had felt some kind of problems with the records; most of them reported poor handwriting, lack of documentation (especially name and family name of patient and date) and also missing of some sheets. They mainly complained of bad handwriting of physicians.

Both physicians and nurses believed that the structure of the PBMR is accurate and there is no need to change it fundamentally, but 50% of physicians and 20% of nurses suggested that to get information easily and quickly it would be better if some predefined tables or checklists could be designed and be filled in only by check marks.

In response to the question "Have you ever requested previous Medical Records of your patients and have you always been successful?" all physicians had requested their patients' previous Medical Records, especially when they had a patient with previous history of hospitalization. If the patient had been hospitalized at the same hospital and/or if she holds a record unit number, it would be easy to retrieve the previous records and in most cases they had access to her previous Medical Records, but if she had been hospitalized at other hospitals, or referred from out patient clinics, the chance of getting access to previous records was very limited.

Ninety percent of the interviewed physicians and Eighty percent of nurses believed that the medical and nursing students should receive additional training about documentation of Medical Records and some of them suggested holding of workshops for physicians and specialists to inform them about how to better document the Medical Records.

Almost all of the physicians believed that those parts of record such as physician's order sheet which are important from the legal aspect, are documented completely and little attention is paid to other parts.

## Discussion

In this study, the availability, completeness and ease of use of the Paper-based Medical Records at a university hospital in Iran has been studied. By considering the results, it is clear that almost all Medical Records in this hospital were incomplete in one way or another. This study revealed that poor handwriting, lack of documentation of requested information and missing of sheets are prominent problems with PBMR in this hospital. This finding is in line with earlier evaluations where incomplete Medical Records have been considered as a major problem [8].

Poor handwriting was so prevalent that in some sheets (especially those sheets which had been filled in by physicians) retrieving of information was almost impossible. In general poor/bad handwriting seemed to be more prevalent among physicians than nurses. In some sheets, the content had been typed (such as laboratory, Radiology and Pathology report sheets), and these sheets were more readable. It indicates that if a machinery system was in place (i.e. EMR system) that could probably improve quality of Medical Records in terms of readability.

Skipping of documentation of information was another prominent finding. Different factors were involved in that problem. Physicians mainly stressed on therapeutic issues so they seemed to have paid more attention on documentation of those parts that have a direct relation with the treatment (such as the medical history and physical exam, physician's order sheet and progress note sheet), and ignored to review other parts of the records to be sure if the information exist. But for nurses, high workload is the main reason for incompleteness of sheets. Nurses believed that most of their working hours were spent on documentation tasks and on some days when workload was high, skipping of documentation of some parts was inevitable [9]. Many of them suggested that an automatic system in which the repeated information (i.e. headers and footers of the sheets) is documented automatically could facilitate their documentation tasks.

On some sheets (mainly essential sheets' group), documentation of treatment provider's identification information, including name, signature and seal of physicians and also name and signature of nurses were near 100% complete. It indicates that physicians and nurses have paid more attention to legal aspect of documentation in order to protect themselves if some jurisdiction issues might happen.

Missing sheets is also another finding of the present study. It is possible that a patient be visited by different residents during afternoon and night shifts if the patient's clinical condition required it. The residents usually do search in the Medical Records for some information (for instance the progress report sheet) for evaluating effectiveness of the current treatment, in that situations missing sheets might affect their decision on continuation or termination of the treatment. Although missing of some sheets in every paper-based systems (not only the PBMR system) is inevitable, but missing sheets at the hospital also indicates that the control and review mechanisms were inadequate, at least in the wards, the head nurses are responsible for reviewing every Medical Record.

With prolongation of hospitalization, one problem becomes prominent "retrieving of information" especially when the record gets bulky [10]. Physicians complain that in bulky records, finding treatment pathway during hospitalization time is difficult. Because of the nature of the problems with the PBMR, it seems that using an EMR system might be useful in solving some parts of the problems (by avoiding of missing sheets and providing quick summary of Medical Records) [11].

The role of the hospital's medical record department on the completeness of records is not deniable [12]. The Medical Record of every patient is transferred to medical records department when the patient is discharged form the hospital. Authorities at the department of the Medical Records are responsible for reviewing and fixing of shortcomings of the records. The results of our study unveiled that some of the Medical Records have been archived without fixing of missing sheets and/or incompleteness of their sheets. We can conclude that the control mechanism at this department was ineffective.

What would happen if the present incomplete PBMR system continues to be used at the hospital? We found that the retrieval of information from the Medical Records at this hospital is a major and time consuming problem. If physicians are not able to find needed information from the Medical Records, they will most probably try to repeat examinations and lab tests, which in both cases would waist time and money for the hospital, physician and the patient. The Medical Records are not only used for treatment purposes, but are also considered as a source of information for research and education. At the university hospitals, physicians are also responsible for conducting research, and sometimes the Medical Records are the only source of information, and incomplete Medical Records could affect the results of the researches.

Inappropriate documentation of Medical Records have been identified as potentially influencing the medical care in a negative way [13], so improving the Medical Records system by using new technologies, for instance EMR system, would probably improve quality of medical care. But we should also be aware of that the same persons would also be responsible to fill in the electronic records and if they don't pay enough attention on documentation process, the similar problems would remain [14,15]. However, the EMR system might also remind the medical staff if important data is missing. The authors are planning to conduct a study on the quality of the Medical Records after introducing of an EMR system at the hospital and compare the results to find out the impact of the EMR system on the quality of the Medical Records.

Finally, it seems that there is a gap between what physicians and nurses have learned and what they do in practice in terms of Medical Records. To solve this problem, they should attend workshops or courses after graduation to get more training and information about the importance of complete Medical Records.

## Conclusion

Due to the low grade of completeness, availability and usability of the Medical Records in this study, it is believed that physicians and nurses at this hospital were not aware of the importance of the Medical Record as a crucial document for treatment and follow-up of their patients. Although the Paper-based Medical Records system might be more effective at bedside and can not be totally eliminated in the near future, it is necessary to find ways to ensure that the documentation of information will be in a readable and retrievable format. For this purpose, predefined tables and forms might improve the situation.

However, the potential of introduction of Electronic Medical Records system with reminders to physicians and nurses when an important data is missing should be studied in the future.

## Competing interests

The authors declare that they have no competing interests.

## Authors' contributions

FP contributed to conception and design of study, design of checklists and interview guidelines, interview with physicians and nurses, quality control of data, analysis and interpretation of data, and drafting of the manuscript. HM involved in developing of study, analysis and interpretation of data and reviewing of the manuscript. AK involved in propagating, analyzing data and reviewing of the manuscript. JE contributed to data analyzing and reviewing of the manuscript. UF contributed to develop of study, analysis and interpretation of data, reviewing and finalizing the manuscript.

## Pre-publication history

The pre-publication history for this paper can be accessed here:


